# Using Short-Form Videos to Get Clinical Trial Newcomers to Sign Up: Message-Testing Experiment

**DOI:** 10.2196/49600

**Published:** 2024-08-15

**Authors:** Sisi Hu, Ciera E Kirkpatrick, Namyeon Lee, Yoorim Hong, Sungkyoung Lee, Amanda Hinnant

**Affiliations:** 1 School of Journalism and Strategic Media University of Arkansas Fayetteville, AR United States; 2 College of Journalism & Mass Communications University of Nebraska-Lincoln Lincoln, NE United States; 3 Department of Mass Communication University of North Carolina at Pembroke Pembroke, NC United States; 4 School of Journalism University of Missouri Columbia, MO United States

**Keywords:** clinical trial recruitment, TikTok, source credibility, framing, psychological barriers, logistical barriers, integrated behavioral model, short-form videos, social media use, clinical trial, recruitment

## Abstract

**Background:**

Recruiting participants for clinical trials poses challenges. Major barriers to participation include psychological factors (eg, fear and mistrust) and logistical constraints (eg, transportation, cost, and scheduling). The strategic design of clinical trial messaging can help overcome these barriers. While strategic communication can be done through various channels (eg, recruitment advertisements), health care providers on the internet have been found to be key sources for communicating clinical trial information to US adults in the social media era.

**Objective:**

This study aims to examine how communication source (ie, medical doctors and peers) and message framing of TikTok videos (ie, psychological and logistical framing) influence clinical trial–related attitudes, perceptions, and sign-up behaviors under the guidance of the integrated behavioral model.

**Methods:**

This study used a 2 (source: doctor vs peer) × 2 (framing: psychological vs logistical) between-participant factorial design web-based experiment targeting adults in the United States who had never participated in clinical trials (ie, newcomers). A Qualtrics panel was used to recruit and compensate the study respondents (n=561). Participants viewed short-form videos with doctors or peers, using psychological or logistical framing. The main outcome measures included perceived source credibility, self-efficacy, attitude toward clinical trial participation, behavioral intention, and sign-up behavior. Structural equation modeling was used to analyze the direct and indirect effects of message factors on the outcome variables. Source (doctor=1; peer=0) and framing (psychological=1; logistical=0) were dummy-coded.

**Results:**

Doctor-featured messages led to greater perceived source credibility (β=.31, *P*<.001), leading to greater self-efficacy (95% CI 0.13-0.30), which in turn enhanced behavioral intention (95% CI 0.12-0.29) and clinical trial sign-up behavior (95% CI 0.02-0.04). Logistical barrier–framed messages led to greater self-efficacy (β=–.09, *P*=.02), resulting in higher intention to participate in clinical trials (95% CI –0.38 to –0.03) and improved sign-up behavior (95% CI –0.06 to –0.004). Logistical barrier–framed messages were also directly associated with an increased likelihood of signing up for a clinical trial (β=–.08, *P*=.03). The model accounted for 21% of the variance in clinical trial sign-up behavior. Attitude did not significantly affect behavioral intention in this study (β=.08, *P*=.14), and psychological and logistical barrier–framed messages did not significantly differ in attitudes toward clinical trial participation (β=–.04, *P*=.09).

**Conclusions:**

These findings advance our understanding of how people process popular message characteristics in short-form videos and lend practical guidance for communicators. We encourage medical professionals to consider short-form video sites (eg, TikTok and Instagram Reels) as effective tools for discussing clinical trials and participation opportunities. Specifically, featuring doctors discussing efforts to reduce logistical barriers is recommended. Our measuring of actual behavior as an outcome is a rare and noteworthy contribution to this research.

## Introduction

### Background

The development and advancement of medical treatments rely heavily on the willingness of individuals to participate in clinical trial research. However, clinical trials have historically had low enrollment rates, especially among underrepresented groups [1,2]. Research aimed at increasing willingness to participate in clinical trials has examined several factors impeding participation [3,4], including a lack of access [4,5], psychological barriers (such as issues related to fear or a lack of trust in research, doctors, and the process), and a lack of understanding of the process and value of clinical trials [6]. Others are deterred by logistical barriers, including financial constraints, time commitment, travel difficulties, and interference with other obligations (eg, work and family) [3,4]. Each barrier requires different solutions and communication strategies to effectively increase overall clinical trial participation [3]. For instance, Clark et al [3] explain that providing compensation, transportation, and flexible hours can help to lessen time and resource-related constraints (ie, logistical barriers); whereas barriers related to fear and a lack of trust (ie, psychological barriers) can be alleviated by explaining the voluntary nature of participation, emphasizing participant safety, and clarifying the participation process. Such actionable recommendations require adjustments by trial administrators (eg, ensuring compensation or help with scheduling and transportation), but also have important communication implications as the information related to overcoming the barriers must be shared with prospective participants.

While communication to prospective participants can be done through various channels (eg, recruitment ads), health care providers on the internet have been found to be critical sources for communicating clinical trial information to US adults [7]. Social media use has saturated society, with 70% of the US population using at least one form of social media [8]. More specifically, short-form video has widely been used for social media apps such as TikTok, a video sharing social media app that allows users to create and host short video content (the worldwide number of TikTok users doubled from 291.4 million in 2019 to 655.9 million in 2021) [9]. Short-form videos, typically lasting from a few seconds to a few minutes, encompass a wide variety of entertaining content, including comedy, dance, music, cooking, fitness, and other daily activities [10]. With social media skyrocketing in popularity, short-form videos are believed to have public health implications as people use TikTok, specifically, to disseminate health-related content [11]. This includes many conversations related to clinical trial research. A May 2023 search indicates that since the platform’s worldwide launch in 2018, the hashtags “#clinicaltrial” and “#clinicalresearch” have received 43.4 million and 22.4 million views, respectively. TikTok has made it increasingly cost-effective for communicators of all types to disseminate information, as anyone with a smartphone can easily record and edit videos directly in the application.

### Communication Source

The most popular sources communicating health information on TikTok are health care professionals (eg, doctors and clinical researchers) and general TikTok users [12,13]. Doctors and other health care professionals have used the platform to cover a variety of topics, including COVID-19, vaping, chronic pulmonary obstructive disease, and diabetes [11-13]. On the topic of clinical trials, doctors on TikTok have shared information on topics such as finding trials [14], upcoming trials [15], the importance of trials [16], and what the participation process is like [17]. Much of the content aims to help potential participants feel more comfortable. For instance, Dr Donald Garcia, medical director at Austin Clinical Trial Partners, explains in his video that institutional review boards are in place to help keep participants safe [17].

Likewise, some general TikTok users have taken to the platform to share their firsthand experience of being in a clinical trial themselves. Some record themselves within the actual clinical trial setting to provide an inside look at what the environment and procedures are like [18], while others detail their experience from home and sometimes answer questions that other users have about the experience [19]. In many cases, these videos advocate for others to participate, too. This sometimes includes the participants sharing details of the monetary compensation they have made from clinical trials and encouraging others to participate for this reason [20]. Testimonials from TikTok users serve as peer-to-peer exchanges of experience-based health information.

The effectiveness of these 2 dominant source types is likely to depend on how the audience perceives each source’s credibility. McCroskey and Teven [21] suggested that 3 key components comprise credibility: competence, trustworthiness, and goodwill. As prior clinical trial participants have firsthand experience with participation, they may be perceived to have high trustworthiness and believability, which are both important for message acceptance [22]. Furthermore, as peer-type sources have similarities to the audience, this can also help to improve message perceptions and effectiveness [23].

Doctors, on the other hand, will likely generate high levels of perceived credibility because of their extensive education and experience (ie, competence). Merely having a respected, authoritative title of “Dr,” alone, can lead others to respect them and listen to what they have to say [24]. Besides their competence, health care providers have been found to be the most trusted source of information about clinical trials in comparison to government health agencies, health organizations, and others such as support groups, drug companies, families, and friends [1]. In addition, research examining the effects of various sources communicating general health information via health podcasts showed that doctors were found to elicit greater perceived source competence and trustworthiness in comparison to peers (individuals with firsthand experience with the health topic) [25]. In addition to competence and trust, doctors and peers discussing clinical trials on TikTok may also generate perceptions of goodwill. Sharing information on TikTok is voluntary, which may help users feel that the doctors and peers have positive intent, truly wanting to help and care for others.

Altogether, given doctors’ high levels of competence (relative to peer sources) and their likelihood of generating perceptions of trust and goodwill, this study predicts that clinical trial messaging on TikTok coming from doctors will result in greater perceptions of source credibility than similar messages that come from general TikTok peers (hypothesis 1 [H1]): doctor-featured messages will lead to greater perceived source credibility toward clinical trial participation than peer-featured messages.

Attitude change can occur through the peripheral route or central route based on individuals’ levels of ability and motivation to think (elaboration likelihood model [ELM] [26]). Source credibility has been found to influence attitude through the peripheral route because it is easy to process [27]. In the case of doctors on TikTok, their credibility (conveyed through their title, introduction, appearance, etc) can serve as peripheral cues (eg, “experts are generally correct”) that improve message acceptance [26]. Since perceived credibility affects message processing and message acceptance [22,28], doctor-featured messages are also expected to cultivate improved audience attitudes and perceptions of self-efficacy. Generally, people rely on sources they trust and reject information from sources they do not trust [29], and acceptance of content influences one’s behavior [30]. Thus, if the source is more credible, individuals will be more likely to perceive the recommended health behavior from the source as effective and feasible. Within the context of TikTok and health content, Song et al [13] found that the information quality of peer TikTok videos was low and engagement (eg, likes, shares) with health professional videos was high. Based on the findings, they suspected that the health professionals conveyed a greater level of expertise that then generated greater credibility perceptions, which could, in turn, improve the adoption of the message recommendations. Research has also shown that increased source credibility leads to favorable attitudes [31] and increased self-efficacy [32] about suggested health behavior. Based on these findings, we hypothesize that as the doctor-featured clinical trial messages increase perceptions of credibility, this will lead to more favorable attitudes and increased self-efficacy (hypothesis 2 [H2]): Doctor-featured messages will lead to (1) more favorable attitudes and (2) greater self-efficacy toward clinical trial participation than peer-featured messages through increased source credibility.

Then, as attitude and self-efficacy are larger drivers of behavioral intention (integrated behavioral model [IBM] [33,34]), the increased attitude and self-efficacy (generated by the doctor-featured messages) will lead to greater intentions to participate in a future trial (hypothesis 3 [H3]): Doctor-featured messages will lead to greater behavioral intention toward clinical trial participation than peer-featured messages through increased (1) attitudes and (2) self-efficacy.

### Message Framing

Within the clinical trial messaging produced by both doctors and prior clinical trial participants, there is an opportunity to emphasize particular information (eg, information about the benefits of participating and the safety of participating). Such emphasis is a form of message framing and is a means of making particular information salient to the audience [35-37]. Framing has been recommended as a strategy for helping to overcome barriers associated with clinical trial participation [3,38,39]. In specific, emphasis framing—which involves designing messages to focus on particular subsets of information related to an overall topic [40]—has been recommended to help prospective participants see how relevant barriers can be overcome (eg, emphasizing safety precautions may help reduce psychological barriers to participation) [3]. Framing can help improve message processing by providing a simplified structure focused on the most important information [41,42]. This leads the audience to focus on and better remember the emphasized information [43,44]. Thus, in the case of clinical trial messaging, when the emphasis is on information that reduces concerns related to the common barriers, the audience will be more likely to process this information, which can then lead to the message favorably affecting their attitudes and behaviors [45]. In addition to having a more favorable attitude toward participation, their self-efficacy for participating should be improved as well, given that the emphasized information provides solutions to the barriers, which should help the barriers seem like less of a deterrent.

In this study, the TikTok messaging is designed to make salient information related to overcoming psychological and logistical barriers. Half of the messages talk about clinical trials with message frames focusing on overcoming psychological barriers, highlighting participants’ well-being and safety during a clinical trial to help prospective participants feel more comfortable about the process (per recommendations from Clark et al [3]). Meanwhile, the logistical barrier–framed messages use the suggestions of Clark et al [3] of focusing on overcoming common logistical issues that impede participation (eg, transportation, scheduling, and finances) to help prospective participants envision how joining a trial could be possible for them [3]. That said, research investigating framing in this context is still new. Logistical barrier–framed messages have not been tested in this manner, and TikTok is still a new, understudied media platform. Specifically, with a diverse sample of individuals who have never participated in a trial before, it is not clear which barrier will be more relevant to them, and thus which barrier-framing will be more effective. Psychological and logistical barrier–framed messaging may be equally effective at improving attitudes and self-efficacy related to clinical trials, or 1 of the 2 framing types might have greater persuasive influence. To explore this, we ask the following question (research question 1 [RQ1]): How does framing (focused on psychological barriers vs logistical barriers) influence (1) attitudes and (2) self-efficacy toward clinical trial participation?

If framing influences attitudes and self-efficacy, the framing may also affect intentions related to participation (through attitudes and self-efficacy) given the relationship between attitudes, self-efficacy, and behavior (IBM [33,34]). Thus, we also ask the following question (research question 2 [RQ2]): How does framing influence intention toward clinical trial participation through (1) attitudes and (2) self-efficacy toward clinical trial participation?

### Pathways to Sign-Up Behavior

In addition to behavioral intention, in this study, we aim to also measure the actual behavior of our participants—whether or not they choose to be redirected (after the study) to a page where they can sign up to participate in future clinical trials. Measuring an actual behavior is valuable because it helps to better understand the persuasive effect of the messages.

While IBM focuses heavily on the formation of behavioral intention, it also explains the importance of environmental constraints and an individual’s knowledge or skills (related to behavior performance) in impacting actual behavior change [46,47]. Environmental constraints are contextual factors that either help or hinder behavior performance [47]. Fishbein and Yzer [48] suggested that health educators should evaluate the potential effectiveness of addressing underlying beliefs or tackling environmental constraints, and then utilize the most appropriate one for the situation. For example, a prospective participant may not intend to participate in a trial because of their beliefs that trials are unsafe (eg, psychological barriers). In this case, a message strategy should be devised to focus on improving the factors influencing intention (eg, attitudes toward the behavior). On the other hand, if a prospective participant has the intrinsic motivation to participate in a trial but might encounter an environmental constraint like transportation issues (eg, logistical barriers), then an effective message strategy is to focus on addressing how to overcome the environmental constraint. In this case, by removing environmental constraints, the performance of the behavior (ie, clinical trial participation) is more likely [49].

Therefore, in this context, there may be indirect pathways in which the source and framing of the messages influence willingness to sign up for a future clinical trial. For instance, a doctor-featured message may increase perceived source credibility which may then improve attitude and self-efficacy which may then improve behavioral intentions and ultimately their behavior. Additionally, there may also be a direct effect of environmental constraints (ie, the contextual factors hindering the behavior) on behavior. For instance, if an individual does not have the literal means (eg, transportation) to get to a clinical trial, they will not participate. Based on this, we predict that our logistical barrier–framed messages (which provide information about overcoming common logistical issues) may have a direct effect on participants’ behavior.

To explore the indirect pathways, we ask the following question (research question 3 [RQ3]): What are the indirect pathways that source and framing influence signing up for a clinical trial?

To examine the direct pathway to sign-up behavior, we pose the following hypothesis (hypothesis 4 [H4]): Logistical barrier–framed messages will lead to a greater likelihood of signing up for a clinical trial than psychological barrier–framed messages.

### Combined Effects of Communication Source and Message Framing

Previous research has examined the interaction between communication sources and message framing [31,50,51]. However, these studies primarily focused on gain and loss frames (or relatedly, positive and negative frames), and the results were inconsistent. For example, Jones et al [31] found the expert source or positive-framed messages generated more behavioral intention than other conditions (eg, nonexpert/positive), whereas Borah and Xiao [50] and Huang and Liu [51] did not find significant interaction effects between framing and source. Considering the novelty of our study, which examines psychological and logistical barrier–framed messages for the first time, it is unclear which barrier frame would be more effective and how it interacts with the message source. Therefore, we ask the following question (research question 4 [RQ4]): How do framing (focused on psychological barriers vs logistical barriers) and message source (doctor vs peer) jointly influence attitudes and self-efficacy, subsequently affecting behavioral intention and sign-up behavior regarding clinical trial participation?

### Study Overview

**Figure 1:**
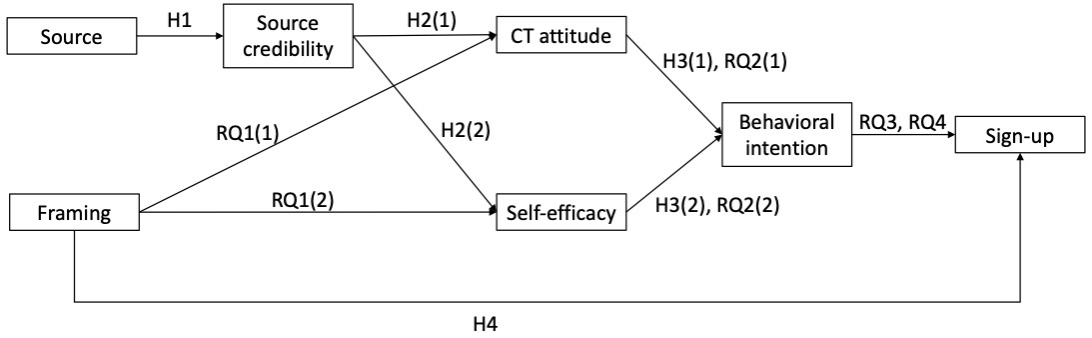
Conceptual framework. CT: clinical trial; H1: hypothesis 1; H2: hypothesis 2; H3: hypothesis 3; H4: hypothesis 4; RQ1: research question 1; RQ2: research question 2; RQ3: research question 3; RQ4: research question 4.

This study aims to examine the effects of short-form video’s message features on clinical trial–related attitudes and behaviors. We test the effects of the 2 communication sources, doctors and peers, commonly seen on TikTok and other social media, as well as the effects of discussing 2 different barriers to clinical trials (psychological and logical framing) in the videos. Informed by the integrated behavioral model [34], the ELM [26], and framing [36,37], this study uses structural equational modeling to test our predictions: The source and message framing of videos indirectly influence behaviors related to clinical trial participation through attitude and self-efficacy, and message framing will directly affect signing up for a clinical trial. We also capture the actual behavior of our participants’ willingness to join a sign-up list for a clinical trial and be contacted by clinical researchers about upcoming clinical participation opportunities. The inclusion of such an outcome is novel and allows us to avoid relying solely on behavioral intention as an indicator of actual behavior outcomes within this context. Figure 1 illustrates the conceptual framework for the above-proposed hypotheses and research questions.

## Methods

### Experimental Design

This study used a 2 (source: doctor vs peer) × 2 (framing: psychological vs logistical) between-subject factorial design web-based experiment targeting adults in the United States who have never participated in clinical trials. A total of 4 conditions were formed (ie, doctor source/psychological barrier–framed, doctor source/logistical barrier–framed, peer source/psychological barrier–framed, and peer source/logistical barrier–framed). For each condition, 3 messages—featuring 3 different clinical trial topics (ie, sleep, stress, and caffeine)—were used to account for message variance [52].

### Respondents and Procedure

Participants were eligible for this study if they were aged 18 years or older and had no prior clinical trial experience. A Qualtrics panel was used to recruit and compensate the study respondents in June 2022. The questionnaire began with informed consent, demographics (ie, age, gender, education, income, political ideology, and race), a question regarding their prior clinical trial participation, and a video test to ensure respondents could see and hear videos. After passing the video test, respondents answered questions measuring their preexisting attitude toward clinical trial participation and were then randomly assigned to 1 of the 4 experimental conditions where they viewed 3 TikTok videos in that condition. The presentation order of the 3 videos was fully randomized to eliminate response bias related to the order of the video presentation. After each of the 3 videos, respondents answered questions checking the manipulation and measuring source credibility and attitudes toward clinical trial participation. After viewing all 3 videos, respondents answered questions measuring self-efficacy toward clinical trial participation and intention to participate in a clinical trial. Last, an IRB-approved fabricated scenario was presented, and respondents were asked to choose whether they were willing to sign up to join a clinical trial participant list and be contacted by clinical researchers about upcoming clinical trial participation opportunities right after this survey. After answering this question, respondents saw the debriefing statement. In the questionnaire, items associated with the same variable were randomized to prevent order-related bias. Each questionnaire page contained between 1 and 5 questions, with a maximum of 67 pages (screens) a respondent could view. Respondents could not review or change their answers during the survey, but they could withdraw their responses at any time.

Qualtrics panel experts handled visitor tracking, participation rates, completion rates, cookies, and IP address duplication checks. The authors double-checked the final data received from the Qualtrics panel. After removing the low-quality responses (n=7), which included speeders (respondents who spent less than half-median amount of time on the survey based on Qualtrics panel expert criteria in the industry), straightliners (respondents who consistently gave identical responses, such as all “7” or “4,” to a series of questions using the same response scale), and respondents with missing data, a total of 561 respondents were used in the data analysis (doctor source/psychological barrier–framed=144, doctor source/logistical barrier–framed=139, peer source/psychological barrier–framed=144, and peer source/logistical barrier–framed=134).

### Stimuli and Manipulation

The stimuli were TikTok videos recorded by 3 female actors on 3 different clinical trial topics (ie, sleep, stress, and caffeine). Each actor focused on one topic and recorded a video for each of the 4 experimental conditions. Each video includes 5 shots: introduction, clinical trial topic overview, mention of barriers, overcoming the barriers, and recommendations. Each shot varied to reflect the 4 experimental conditions. The key messages were also added as captions to each video. All other video features were kept similar across the 4 experimental conditions, including the video length (around 90 seconds), TikTok logo, and caption color and font. All 12 transcripts were edited by a professional news editor, and all 12 videos were reviewed by the research team. Examples of message transcripts are provided in Multimedia Appendix 1.

The source referred to the type of speakers in the video, including doctor source (ie, clinical researcher) and peer source (ie, prior participant). In the doctor-featured videos, actors wore professional white lab coats in an office setting with a blank wall background, while in the peer-featured videos, actors wore casual clothes in a home setting. Specifically, in each doctor-featured message, the actor introduced herself with the “Dr” honorific and as a clinical trial researcher and then brought up the clinical trial topic she worked on. She then talked about the barriers people may have in terms of clinical trial participation (ie, psychological or logistical) and how clinical researchers attempt to address the barriers. Last, the actor recommended that the audience sign up for clinical trials and reiterated that they attempted to address the barriers. In a peer-featured message, the actor introduced herself as a clinical trial participant and then shared her experience of clinical trial participation. She started by mentioning her barriers before clinical trial participation, and then she talked about how her barriers had been resolved during the clinical trial participation. Finally, the actor recommended the audience sign up for clinical trials and reiterated the clinical researchers’ attempts to address the barriers.

Framing referred to the type of barrier information made salient in the post, including psychological barriers and logistical barriers. Psychological barrier–framed videos focused on addressing psychological barriers to participating in clinical trials (eg, fear about clinical trial participation and medical mistrust) by highlighting participants’ well-being and safety during a clinical trial. Logistical barrier–framed videos centered on overcoming logistical barriers to clinical trial participation (eg, cost and flexibility) by emphasizing the monetary reward and flexibility of participation. These frame wordings were created based on the suggestion from Clark et al [3]. The framing was manipulated in the mention of barriers, overcoming the barriers, and recommendations sections in the videos. A manipulation check was performed to ensure the 2 framing categories were distinguished in the videos. After each video, respondents were asked to indicate what the video emphasized (1=participants’ well-being and safety, 5=Monetary reward and flexibility of participation). An independent samples *t* test showed a significant difference between the psychological barrier–framed videos (mean 1.54, SD 0.84; n=288) and the logistical barrier–framed videos (mean 3.88, SD 0.94; n=273), *t*_545.55_=−31.04 (2-tailed), *P*<.001. Thus, the psychological barrier–framed videos were perceived as emphasizing participants’ well-being and safety more whereas the logistical barrier–framed videos were perceived as emphasizing monetary reward and flexibility of participation more.

### Measurements

Preexisting attitude toward clinical trial participation assessed respondents’ positive or negative feelings toward clinical trial participation before exposure to the stimuli. Respondents were asked to rate the degree of their perception of clinical trial participation on a 5-item, 5-point semantic differential scale adapted from Kang and Lee [53], such as bad/good and negative/positive. (across the conditions, Cronbach α=0.87-0.92.)

Perceived source credibility assessed respondents’ perceptions of the video speaker’s competence, goodwill, and trustworthiness with an 18-item scale adapted from McCroskey and Teven [21]. Respondents were asked to rate their perception of the speaker on a 5-point bipolar scale, including items such as untrained/trained, cares about me/does not care about me, and untrustworthy/trustworthy (across the conditions, Cronbach α=0.96-0.98).

Self-efficacy measured respondents’ belief in their capabilities to participate in clinical trials with a 5-point Likert scale of 2 items adapted from Lee et al [54]. Items included, “for me, to participate in a clinical trial would be difficult (1) to easy (5)” and “How certain are you that you could participate in a clinical trial? (1=Not at all certain; 5=Very certain)” (across the conditions, Cronbach α=0.82-0.86).

Attitude toward clinical trial participation assessed respondents’ positive or negative feelings toward clinical trial participation after clinical trial to the stimuli with the same scale used to measure preexisting attitudes toward clinical trial participation. (across the conditions, Cronbach α=0.96-0.97).

Behavioral intention assessed the likelihood that respondents would participate in clinical trials with a 5-point Likert scale of 2 items adapted from Chen et al [55]. Respondents were asked to rate their opinion on 2 items from strongly disagree (1) to strongly agree (5), including “I plan on joining a clinical trial” and “I am willing to join a clinical trial” (across the conditions, Cronbach α=0.83-0.91).

Sign-up behavior was measured by whether the respondents clicked “Yes” to move forward to a sign-up page to participate in future clinical trials. Although this question did not measure participants’ behavior beyond clicking “Yes” to share their email address, the behavior of choosing to go to the sign-up page is a reasonably accurate indicator for actual sign-up behavior (Yes=45.6%, 256/561). After participants answered this question, they were not directed to a sign-up page. Instead, they saw a debriefing statement that explained the true purpose of this question, and they were given information on how to find clinical trial participation options.

### Ethical Considerations

This study received approval from the institutional review board of the University of Missouri (IRB #2054423). Informed consent was obtained from participants at the beginning of the survey. The consent form explained that the survey would take approximately 15-20 minutes, emphasized voluntary and anonymous participation, assured participants that their data would be stored securely in a password-protected electronic format, and clarified that researchers would only report aggregate data. Participants who did not agree with the consent form had the option to opt out. A Qualtrics panel was used to recruit and compensate the study participants at the agreed-upon rate established between the participants and Qualtrics.

### Statistical Analysis

The hypothesized model was tested using structural equation modeling with the lavaan package for R [56]. All models were estimated using robust maximum likelihood unless bootstrapping was used, in which case ML estimation was adopted. Robust maximum likelihood was used because this model includes a binary outcome variable (ie, sign-up) [57]. Following Kline’s (2015) 2-step process, a measurement model was first fit to verify the factor structure of clinical trial attitude, efficacy, and behavioral intention. Subsequently, to test the hypotheses, a structural model was fit in which source credibility, clinical trial attitudes, and self-efficacy were regressed on dummy-coded message source (doctor=1, peer=0), framing (psychological barrier frame=1, logistical barrier frame=0), and the interaction term of source and framing. The behavioral intention was regressed on clinical trial attitudes and self-efficacy. Sign-up behavior was regressed on behavioral intention, message source, framing, and the interaction term of source and framing. All variables in the model were regressed on the control variable (ie, preexisting attitudes toward clinical trial participation). To maintain the model simplicity, however, the interaction term of source and framing was omitted from the final model as it did not exert any significant effect.

## Results

### Sample Overview

The average age of the sample was 49 (SD 17.52) years, and there were more females (301/561, 53.7%) than males (256/561, 45.6%) and 4 participants did not disclose. Respondents were primarily White (433/561, 77.2%), followed by Black or African American (52/461, 9.3%), Asian (39/561, 7%), and others (30/561, 5.3%). Approximately half of the respondents completed high school or some college (286/561, 51%), 13.7% (n=77) respondents had an associate’s degree, and 32.8% (n=184) respondents had a bachelor degree or above. From liberal (1) to conservative (7), the sample leaned conservative (mean 3.93, SD 1.59).

### Modeling Results

**Figure 2:**
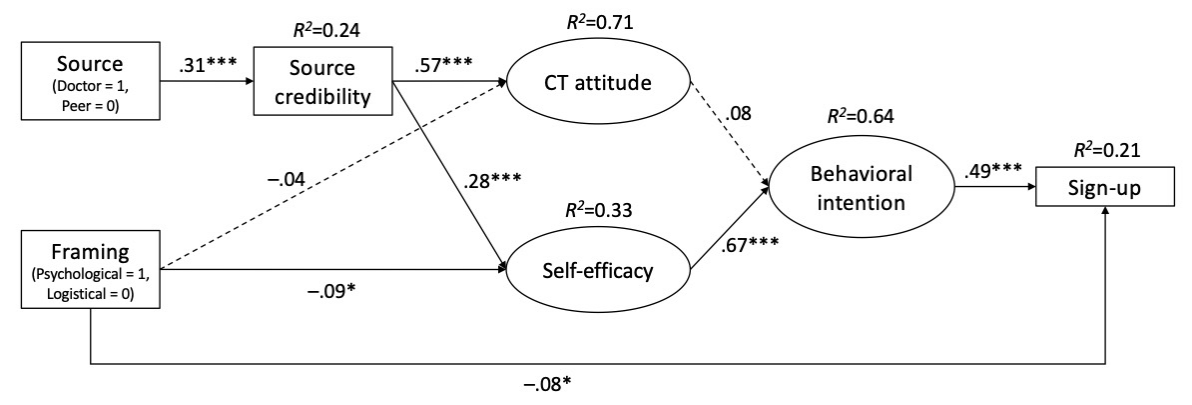
Final structural model (N=561). The model controlled for preexisting attitudes toward clinical trial participation. Path coefficients are standardized (β). Dashed lines represent nonsignificant paths. *P<.05, **P<.01, and ***P<.001 (specific *P* values are reported in the results texts to minimize repetitive reporting).

Model fit for the measurement model was acceptable based on the criteria from MacCallum et al [58] and Little [59] (*χ^2^*_24_=92.6, *P*<.001); robust root-mean-square error of approximation=0.063 (90% CI 0.079-0.097); robust comparative fit index=0.984; robust nonnormed fit index/Tucker Lewis index=0.975; and standardized root mean residual=0.026. The final structural model (Figure 2) achieved good model fit based on the same criteria as above (*χ^2^*_65_=206.01, *P*<.001); robust root-mean-square error of approximation=0.066 (90% CI 0.056-0.076); robust comparative fit index=0.974; robust nonnormed fit index/Tucker Lewis index=0.965; and standardized root mean residual=0.04.

### Hypotheses Testing

H1 predicted that doctor-featured messages would lead to greater perceived source credibility toward clinical trial participation than peer-featured messages. Consistent with this prediction, compared with peer-featured messages, doctor-featured messages were significantly associated with increased perceived source credibility (β=.31, *P*<.001).

H2 predicted that doctor-featured messages would lead to (1) more favorable attitudes and (2) greater self-efficacy toward clinical trial participation than peer-featured messages through increased source credibility. In support of this hypothesis, there were significant positive indirect effects of doctor-featured messages on clinical trial attitudes (95% CI 0.48-0.84) and self-efficacy toward clinical trial participation (95% CI 0.13-0.30) through the increased source credibility, as evidenced by a 5000 bootstrapped 95% CI that did not contain zero.

H3 hypothesized that doctor-featured messages would lead to greater behavioral intention toward clinical trial participation than peer-featured messages through increased (1) attitudes and (2) self-efficacy. The model showed clinical trial attitudes were not significantly associated with the increased intention to participate in clinical trials (β=.08, *P*=.14); thus, inconsistent with H3(1), doctor-featured messages did not lead to greater clinical trial participation intention than peer-featured messages through increased attitudes. However, self-efficacy was significantly positively associated with clinical trial participation intention (β=.67, *P*<.001); therefore, in support of H3(2), doctor-featured messages led to greater clinical trial participation intention than peer-featured messages through increased perceived source credibility and self-efficacy (95% CI 0.12-0.29).

RQ1 examined the influence of framing on (1) attitudes and (2) self-efficacy toward clinical trial participation. The results showed the effects of psychological barrier–framed messages and logistical barrier–framed messages did not significantly differ in attitudes toward clinical trial participation (β=–.04, *P*=.09). However, logistical barrier–framed messages led to significantly greater self-efficacy toward clinical trial participation than psychological barrier–framed messages (β=–.09, *P*=.02).

RQ2 investigated the indirect effects of framing on the intention to participate in clinical trials through (1) attitudes and (2) self-efficacy. Because different framing foci did not lead to significantly different direct effects on attitudes (shown in RQ1(1)), the indirect effect of framing on intention to participate in clinical trials through attitudes could not be significant (RQ2(1)). For RQ2(2), however, logistical barrier–framed messages led to significantly greater intention to participate in clinical trials than psychological barrier–framed messages through increased self-efficacy (95% CI –0.38 to –0.03).

RQ3 explored the indirect effects of source and framing on sign-up behavior. There are 2 significant pathways. First, doctor-featured messages led to greater perceived source credibility, which, in turn, led to greater self-efficacy and then increased behavioral intention, which, last, boosted the clinical trial sign-up behavior (95% CI 0.02-0.04). Second, logistical barrier–framed messages led to greater self-efficacy, which, in turn, increased intention to participate in clinical trials, which, last, improved the clinical trial sign-up behavior (95% CI –0.06 to –0.004).

H4 posited a direct effect of framing on sign-up behavior, such that logistical barrier–framed messages would lead to a greater likelihood of signing up for a clinical trial than psychological barrier–framed messages. Consistent with this prediction, compared with psychological barrier–framed messages, logistical barrier–framed messages were associated with an increased likelihood of signing up for a clinical trial (β=–.08, *P*=.03).

RQ4 explored the joint effects of framing and sources. There was no significant interaction between these 2 variables, as tested in the modeling section. However, their combined effects were notable. The results revealed that framing and sources independently influenced sign-up behavior. Together, the model accounted for 21% of the variance in the likelihood of signing up for a clinical trial.

## Discussion

### Summary of Findings

This research offers several important findings that illuminate strategic ways to use short-form social media videos, such as those on TikTok, to improve perceptions of clinical trials and increase enrollment. The findings suggested that doctor-featured messages led to greater perceived source credibility, leading to greater self-efficacy, subsequently enhancing behavioral intention and clinical trial sign-up behavior. Logistical barrier–framed messages led to greater self-efficacy, resulting in higher intention to participate in clinical trials and improved sign-up behavior. Logistical barrier–framed messages were also directly associated with an increased likelihood of signing up for a clinical trial. The theoretical and practical implications, limitations, and future directions of this study are discussed below.

### Finding Implications

First, using doctors (ie, clinical researchers) as the source sharing the information made a difference in how participants assessed credibility (H1). The doctors were perceived as more credible than the peer clinical-trial participants, and the credibility afforded to the doctors led to better attitudes about clinical trials and an increased feeling of self-efficacy to engage in clinical trials among the participants (H2). This finding accords with previous ELM research that found higher perceived credibility resulted in enhanced favorable attitudes [22,28]. Extending the path even further, the perceived self-efficacy, cultivated in part by the doctors’ credibility, also led to greater intention to sign up for clinical trials (H3(2)) and ultimately increased clinical trial sign-up behavior (RQ3). This finding confirmed the significant mediating role of self-efficacy in the relationship between source credibility and behavioral intention. Unexpectedly, attitudes were found not to mediate this relationship (H3(1)), supporting the idea that the ELM is more a model of attitude change than persuasion as strong attitudes do not necessarily induce behavioral change particularly if self-efficacy is lacking [60]. Petty et al [61] stressed the importance of attitude change in eliciting behavioral change by explaining that self-efficacy can be “positive attitudes toward the self” (distinct from attitudes toward a behavior) and suggested scholars should identify the most important type of attitudes for predicting a particular health behavior. Despite varying explanations, our findings indicate that future scholars should incorporate self-efficacy into the ELM, either as an additional construct or a critical type of attitude in health communication. It is noteworthy that, as far as the authors are aware, this is the first ELM study that captures persuasive effects on a type of behavioral enactment in the form of clicking to sign up for future clinical trials.

Second, this research contributes to IBM research in that it demonstrated the effects of the messages on attitude, self-efficacy (RQ1), behavioral intention (RQ2), and sign-up behavior (RQ3/H4) on TikTok within the context of health communication. Fishbein and Yzer [48] suggested that changing the psychological determinants to perform a behavior would be more efficient than changing skills and environmental constraints because the latter is often difficult to change. However, this study found that certain environmental constraints, such as transportation issues (ie, logistical barriers), can be communicated and can directly lead to the desired behavioral performance. As IBM affords the flexibility to communicate about a wide range of determinants, a proper IBM-based research agenda could allow communicators to target a health behavior that is stymied by both psychological and logistical barriers.

Third, our findings about framing revealed that the logistical frame outperformed the psychological frame (RQ2(2), H4), which accords with expectations that different frames would lead to different reactions [36,37]. More specifically, framing according to perceived barriers to clinical trials and their solutions, was predicted to have persuasive power [3,38,39]. Regardless of source type, the messages that were framed to address how to overcome logistical barriers had a direct effect on increasing participants’ clinical trial sign-up behavior (H4). This represents an original contribution, given that logistical barrier–framing has not yet been tested. The power of focusing on logistical problems and how to overcome them is promising for both theory and practice.

Finally, despite the lack of interaction between source and framing, the finding demonstrated doctor sourcing and logistical barrier–framing independently but cumulatively contributed to increasing sign-up behavior (RQ4). This finding, along with the abovementioned findings regarding the independent effects of source and framing, suggests that incorporating doctor sources and logistical barrier–framing in health communication, particularly through short-form videos, can yield more successful outcomes in terms of behavioral change.

### Limitations and Future Research

One limitation of this research is that it is difficult to measure full commitment to participate in a clinical trial. We designed the sign-up option to replicate the first behavioral step that a person could take when seeking out clinical trials. It is one of several steps a person would need to take to fully participate in a clinical trial, thereby representing behavior change. Another limitation of this study is the omission of perceived norms toward clinical trials in the integrated behavioral model. This decision assumed that clinical trials are less prevalent compared with other health behaviors, and therefore perceived norms may not exert significant variance. However, it is important to note that exposure to videos featuring prior clinical trial participants may enhance perceived norms by increasing the perception that other people with similar barriers are engaging in the behavior (ie, descriptive norms). Thus, future studies should explore the influence of source and framing on perceived norms and their subsequent influence on behavioral intentions and actual behaviors. Last, it is important to note that the sample in this study mainly consisted of White individuals with a conservative leaning. We suggest that scholars exercise caution when generalizing the current findings to significantly different racial and ideological groups.

Future research should also create similar stimuli to test the same framing and sourcing patterns with different health topics that might involve psychological and logistical barriers, such as blood donation. Furthermore, investigating source and framing preferences among diverse racial and age groups is also essential, given disparities in clinical trial participation within specific demographics [2]. Additionally, future research could examine whether one’s level of familiarity and experience with TikTok (and other related platforms) moderate the effects of these message features.

### Conclusion and Practical Recommendations

Given the effectiveness of the doctor-sourced videos in this study, we recommend that medical professionals take to short-form video sites such as TikTok to discuss clinical trials and participation opportunities. Recent recommendations have advised health professionals to leverage social media platforms like TikTok to disseminate health information [11,13], and this study provides evidence for this recommendation within this specific context of clinical trials. As none of the participants in this research had ever been part of a clinical trial before, this research is especially useful for outreach to people who have likely given clinical trials little thought or who have considered participating but did not follow through. This study mirrors one step in the recruiting process, which is getting potential participants to agree to receive information about future clinical trials. Recruiters could see a similar willingness to sign up for clinical trials if their promotional material involved doctors speaking about how they have worked to reduce logistical barriers. With TikTok users already turning to this platform to share clinical-trial experiences and seek related content (as evidenced by the popularity of the #clinicaltrial and #clinicalresearch hashtags), TikTok may be an especially useful mode of communication. This study serves to inform those interested in taking advantage of this new modality for public health messaging.

## Appendix

Multimedia Appendix 1 Message transcripts for the sleep topic.

## Abbreviations

ELM: elaboration likelihood model
H1: hypothesis 1
H2: hypothesis 2
H3: hypothesis 3
H4: hypothesis 4
IBM: integrated behavioral model
RQ1: research question 1
RQ2: research question 2
RQ3: research question 3
RQ4: research question 4

## Data Availability

The data sets generated or analyzed during this study are available from the corresponding author upon request.
