# Supplementary material for: Using Short-Form Videos to Get Clinical Trial Newcomers to Sign Up: Message-Testing Experiment
Source: J Med Internet Res. 2024 Aug 15;26:e49600. doi: 10.2196/49600 (PMC11362704; doi:10.2196/49600)
Supplement: Multimedia Appendix 1 [file jmir_v26i1e49600_app1.pdf]

Appendix 1: *Message Transcripts for the Sleep Topic*

Source	Shot	Psychological-barrier-frame	Logistical-barrier-frame
Doctor-source<br><br>Account username: @drwilson417	*Introduction*	Hi everyone, for those of you who don't know me, I'm Dr. Wilson. I'm here today to talk about what it's like to be a clinical trial participant. Clinical trials are important because they allow us to develop new treatments for people.<br><br>[Caption: It's Dr. Wilson talking about clinical trials.]	
	CT topic overview	I study sleep-related problems. But I don't just need people with sleep-related problems in my studies. Healthy people are important too so that I can make comparisons between regular people and people with sleep problems.<br><br>[Caption: Sleep-related problems are my focus. Healthy people should sign up, too.]	
	Mention of barriers	Before you sign up for trials, you might not have much information and, to be honest, feel a little nervous around researchers in lab coats. You might not be sure about how much your health and well-being will be taken into account by the researchers.<br><br>[Caption: It's normal to feel nervous! You might be worried about your health and well-being.]	You might be worried that a clinical trial would take time away from work and family. You also might think it costs too much to get to the place where the clinical trial is happening or that the times they want you there won't work for your schedule.<br><br>[Caption: You might worry about the time it takes. Or the cost to get to the trial.]
	Overcoming the barriers	So, what we do is give a lot of information about how we measure your sleep, and we fully answer any questions you might have. During the study, we monitor your health very carefully and pay attention to any related issues from the sleep study.<br><br>[Caption: But we do give you info and monitor you carefully.]	To help relieve financial strain, we pay you well for your time and also cover transportation costs for the few times you need to come to and from the study. We will also make sure it doesn't conflict with your work schedule or when you need to be home for family.<br><br>[Caption: We cover costs, pay you for your time and work with your schedule.]
	Recommend ations	I would recommend that you sign up for a clinical trial about sleep because we will take care of you and make sure you have the information to make good decisions about your health.<br><br>[Caption: Sign up for a clinical trial and know we will keep you informed!]	I recommend that you sign up for a clinical trial about sleep because we will make sure it's beneficial for you without being costly, inconvenient or disrupting your everyday life.<br><br>[Caption: Sign up for a clinical trial and know we will make it worthwhile!]

Source	Shot	Psychological-barrier-frame	Logistical-barrier-frame

Peer-source<br><br>Account username: @amanda8858	*Introduction*	Hey everyone. A few weeks ago, I decided to be a participant in a clinical trial, and today, I thought I'd hop on here and share what my experience was like!<br><br>[Caption: I was in a clinical trial today!]	
	CT topic overview	The clinical trial I was in was testing a treatment for sleep-related problems. I don't actually have sleep problems, but researchers also need healthy people like me to provide data that can be used as a comparison.<br><br>[Caption: The study was about sleep problems Healthy people can sign up, too!]	
	Mention of barriers	Before I got into the clinical trial, I didn't have a lot of information, and to be honest, researchers in lab coats kind of make me nervous. I also wasn't sure how much my health and well-being would be taken into account by the researchers.<br><br>[Caption: Lab coats make me nervous! I was scared about my health while participating.]	I was worried the clinical trial would take up my time that I should spend either at work or home relaxing. I also wasn't sure how much it would cost to get to where the study was taking place or if I could even be there when they wanted me to.<br><br>[Caption: I worried about how long it would take. I also didn't know how I would get there.]
	Overcoming the barriers	It turned out, they gave me a lot of information about how they were going to measure my sleep, and I was able to get answers to all of my questions. The researchers watched my health very carefully and paid attention to any related issues from the sleep study.<br><br>[Caption: But they watched me carefully and answered all my questions.]	I was relieved when they paid me well for my time and also gave me money to cover travel to and from the study, which was only a few times. They let me come in at times that wouldn't mess with my work schedule or when I needed to be home for family.<br><br>[Caption: But they paid me to come and it didn't mess up my schedule.]
	Recommend ations	I would recommend that you sign up for a clinical trial about sleep because the researchers will take care of you and make sure you have the information to make good decisions about your health. Really, everyone, sign up for clinical trials because they are the only way researchers can find new solutions for health problems.<br><br>[Caption: Sign up for a clinical trial — they keep you informed!]	I recommend that you sign up for a clinical trial about sleep because it was not costly or inconvenient — in fact, they make it really easy for you to go about your regular routine during the trial. It's so important people sign up for clinical trials because they are the only way for doctors to find out new ways to help folks out.<br><br>[Caption: Sign up for a clinical trial — they make it worthwhile!]
